# 3-(4-Fluoro­benzyl­idene)-1,5-dioxa­spiro­[5.5]undecane-2,4-dione

**DOI:** 10.1107/S1600536810054395

**Published:** 2011-01-08

**Authors:** Wu-Lan Zeng

**Affiliations:** a MicroScale Science Institute, Department of Chemistry and Chemical Engineering, Weifang University, Weifang 261061, People’s Republic of China

## Abstract

In the title mol­ecule, C_16_H_15_FO_4_, the fused 1,3-dioxane and cyclo­hexane rings exhibit a bath and a chair conformation, respectively. In the crystal, weak inter­molecular C—H⋯O hydrogen bonds link the mol­ecules into centrosymmetric dimers.

## Related literature

For related structures, see: Zeng & Jian (2009 ▶); Zeng *et al.* (2009 ▶). For applications of spiro compounds, see: Jiang *et al.* (1998 ▶); Lian *et al.* (2008 ▶); Wei *et al.* (2008 ▶).
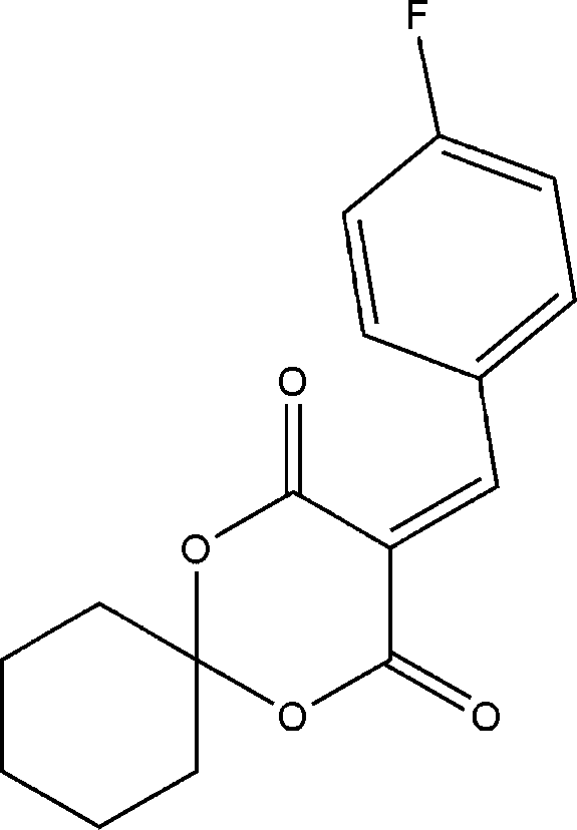


## Experimental

### 

#### Crystal data

C_16_H_15_FO_4_
*M* *_r_* = 290.28
Triclinic, 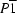

*a* = 5.6690 (11) Å
*b* = 10.130 (2) Å
*c* = 12.160 (2) Å
α = 100.68 (3)°
β = 90.73 (3)°
γ = 91.20 (3)°
*V* = 686.0 (2) Å^3^
*Z* = 2
Mo *K*α radiation
μ = 0.11 mm^−1^
*T* = 293 K
0.25 × 0.18 × 0.12 mm

#### Data collection

Bruker SMART CCD area-detector diffractometer
6753 measured reflections
3124 independent reflections
2481 reflections with *I* > 2σ(*I*)
*R*_int_ = 0.029

#### Refinement

*R*[*F* ^2^ > 2σ(*F* ^2^)] = 0.040
*wR*(*F* ^2^) = 0.127
*S* = 1.11
3124 reflections
190 parameters
H-atom parameters constrained
Δρ_max_ = 0.22 e Å^−3^
Δρ_min_ = −0.22 e Å^−3^

### 

Data collection: *SMART* (Bruker, 1997 ▶); cell refinement: *SAINT* (Bruker, 1997 ▶); data reduction: *SAINT*; program(s) used to solve structure: *SHELXS97* (Sheldrick, 2008 ▶); program(s) used to refine structure: *SHELXL97* (Sheldrick, 2008 ▶); molecular graphics: *XP* in *SHELXTL* (Sheldrick, 2008 ▶); software used to prepare material for publication: *SHELXL97*.

## Supplementary Material

Crystal structure: contains datablocks global, I. DOI: 10.1107/S1600536810054395/cv5023sup1.cif

Structure factors: contains datablocks I. DOI: 10.1107/S1600536810054395/cv5023Isup2.hkl

Additional supplementary materials:  crystallographic information; 3D view; checkCIF report

## Figures and Tables

**Table 1:** Hydrogen-bond geometry (Å, °)

*D*—H⋯*A*	*D*—H	H⋯*A*	*D*⋯*A*	*D*—H⋯*A*
C12—H12*A*⋯O2^i^	0.93	2.47	3.3405 (17)	156

Symmetry code: (i) 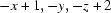.

**Fig. 1.**
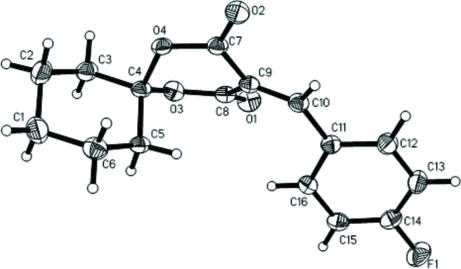
The molecular structure of (I), drawn with 30% probability displacement ellipsoids.

## supplementary crystallographic information

### Comment

Spiro compounds are widely used in medicine, catalysis and optical material (Lian *et al.*, 2008; Jiang *et al.*, 1998; Wei *et al.*, 2008). As a part of our search for new spiro compounds with potentially high bioactivity (Zeng *et al.*, 2009a,b), the title compound, (I), has been synthesized. Herewith we present its crystal structure.

In (I) (Fig. 1), the 1,3-dioxane ring is in a bath conformation with atom C4 atom common to the cyclohexane forming the flap. The cyclohexane ring exists in a distorted chair comformation. In the crystal structure, weak intermolecular C—H···O hydrogen bonds (Table 1) link the molecules into centrosymmetric dimers.

### Experimental

The mixture of malonic acid (6.24 g, 0.06 mol) and acetic anhydride(9 ml) in strong sulfuric acid (0.25 ml) was stirred with water at 303K, After dissolving, cyclohexanone (5.88 g, 0.06 mol) was added dropwise into solution for 1 h. The reaction was allowed to proceed for 3 h. The mixture was cooled and filtered, and then an ethanol solution of 4-fluorobenzaldehyde (7.44g,0.06 mol) was added. The solution was then filtered and concentrated. Single crystals were obtained by evaporation of an petroleum ether-ethylacetate (3:1 *v*/*v*) solution of (I) at room temperature over a period of one week.

### Refinement

The H atoms were placed in calculated positions (C—H = 0.93–0.97 Å), and refined as riding, with *U*_iso_(H) = 1.2*U*_eq_(C).

### Crystal data

**Table:**

C_16_H_15_FO_4_	*Z* = 2
*M**_r_* = 290.28	*F*(000) = 304
Triclinic, *P*1	*D*_x_ = 1.405 Mg m^−^^3^
Hall symbol: -P 1	Mo *K*α radiation, λ = 0.71073 Å
*a* = 5.6690 (11) Å	Cell parameters from 2481 reflections
*b* = 10.130 (2) Å	θ = 3.4–27.5°
*c* = 12.160 (2) Å	µ = 0.11 mm^−^^1^
α = 100.68 (3)°	*T* = 293 K
β = 90.73 (3)°	Block, colourless
γ = 91.20 (3)°	0.25 × 0.18 × 0.12 mm
*V* = 686.0 (2) Å^3^	

### Data collection

**Table:**

Bruker SMART CCD area-detector diffractometer	2481 reflections with *I* > 2σ(*I*)
Radiation source: fine-focus sealed tube	*R*_int_ = 0.029
graphite	θ_max_ = 27.5°, θ_min_ = 3.4°
phi and ω scans	*h* = −6→7
6753 measured reflections	*k* = −13→13
3124 independent reflections	*l* = −15→15

### Refinement

**Table:**

Refinement on *F*^2^	Primary atom site location: structure-invariant direct methods
Least-squares matrix: full	Secondary atom site location: difference Fourier map
*R*[*F*^2^ > 2σ(*F*^2^)] = 0.040	Hydrogen site location: inferred from neighbouring sites
*wR*(*F*^2^) = 0.127	H-atom parameters constrained
*S* = 1.11	*w* = 1/[σ^2^(*F*_o_^2^) + (0.075*P*)^2^ + 0.0414*P*] where *P* = (*F*_o_^2^ + 2*F*_c_^2^)/3
3124 reflections	(Δ/σ)_max_ < 0.001
190 parameters	Δρ_max_ = 0.22 e Å^−^^3^
0 restraints	Δρ_min_ = −0.22 e Å^−^^3^

### Special details

**Table:**

Geometry. All e.s.d.'s (except the e.s.d. in the dihedral angle between two l.s. planes) are estimated using the full covariance matrix. The cell e.s.d.'s are taken into account individually in the estimation of e.s.d.'s in distances, angles and torsion angles; correlations between e.s.d.'s in cell parameters are only used when they are defined by crystal symmetry. An approximate (isotropic) treatment of cell e.s.d.'s is used for estimating e.s.d.'s involving l.s. planes.
Refinement. Refinement of *F*^2^ against ALL reflections. The weighted *R*-factor *wR* and goodness of fit *S* are based on *F*^2^, conventional *R*-factors *R* are based on *F*, with *F* set to zero for negative *F*^2^. The threshold expression of *F*^2^ > σ(*F*^2^) is used only for calculating *R*-factors(gt) *etc*. and is not relevant to the choice of reflections for refinement. *R*-factors based on *F*^2^ are statistically about twice as large as those based on *F*, and *R*- factors based on ALL data will be even larger.

### Fractional atomic coordinates and isotropic or equivalent isotropic displacement parameters (Å^2^)

**Table:**

	*x*	*y*	*z*	*U*_iso_*/*U*_eq_	
O4	0.41354 (13)	0.17850 (9)	0.73171 (7)	0.0412 (2)	
O3	0.23315 (15)	0.01227 (9)	0.59454 (6)	0.0443 (2)	
O2	0.54128 (17)	0.12738 (11)	0.89002 (8)	0.0566 (3)	
O1	0.19126 (18)	−0.19449 (10)	0.62363 (8)	0.0542 (3)	
C8	0.21663 (19)	−0.07730 (12)	0.66337 (10)	0.0397 (3)	
F1	−0.52419 (17)	−0.46737 (10)	0.87835 (8)	0.0718 (3)	
C7	0.41388 (19)	0.09907 (13)	0.80943 (9)	0.0390 (3)	
C11	−0.0075 (2)	−0.18139 (12)	0.86756 (9)	0.0386 (3)	
C9	0.25581 (19)	−0.02211 (12)	0.78410 (9)	0.0369 (3)	
C4	0.23557 (19)	0.15348 (12)	0.64363 (9)	0.0373 (3)	
C15	−0.3686 (2)	−0.29703 (14)	0.79341 (12)	0.0483 (3)	
H15A	−0.4931	−0.3097	0.7417	0.058*	
C10	0.1751 (2)	−0.07673 (12)	0.86916 (10)	0.0395 (3)	
H10A	0.2448	−0.0435	0.9387	0.047*	
C14	−0.3539 (2)	−0.37296 (13)	0.87486 (11)	0.0477 (3)	
C5	−0.00563 (19)	0.19627 (13)	0.68678 (10)	0.0398 (3)	
H5A	−0.0431	0.1530	0.7494	0.048*	
H5B	−0.1233	0.1671	0.6282	0.048*	
C16	−0.1937 (2)	−0.20081 (13)	0.78976 (11)	0.0446 (3)	
H16A	−0.2004	−0.1483	0.7347	0.053*	
C3	0.3168 (2)	0.22760 (16)	0.55360 (11)	0.0510 (3)	
H3A	0.2194	0.1992	0.4869	0.061*	
H3B	0.4783	0.2043	0.5348	0.061*	
C13	−0.1757 (3)	−0.35709 (15)	0.95384 (11)	0.0526 (3)	
H13A	−0.1711	−0.4106	1.0082	0.063*	
C12	−0.0029 (2)	−0.25937 (14)	0.95053 (10)	0.0473 (3)	
H12A	0.1176	−0.2457	1.0043	0.057*	
C6	−0.0155 (2)	0.34786 (14)	0.72405 (12)	0.0522 (3)	
H6A	−0.1749	0.3723	0.7460	0.063*	
H6B	0.0879	0.3758	0.7887	0.063*	
C2	0.3031 (3)	0.37944 (17)	0.59021 (14)	0.0625 (4)	
H2A	0.4182	0.4098	0.6497	0.075*	
H2B	0.3421	0.4226	0.5277	0.075*	
C1	0.0590 (3)	0.42054 (17)	0.63108 (15)	0.0642 (4)	
H1A	−0.0536	0.3997	0.5692	0.077*	
H1B	0.0590	0.5168	0.6584	0.077*	

### Atomic displacement parameters (Å^2^)

**Table:**

	*U*^11^	*U*^22^	*U*^33^	*U*^12^	*U*^13^	*U*^23^
O4	0.0287 (4)	0.0539 (5)	0.0422 (4)	−0.0084 (3)	−0.0059 (3)	0.0136 (4)
O3	0.0462 (5)	0.0525 (5)	0.0318 (4)	0.0047 (4)	0.0000 (3)	0.0016 (4)
O2	0.0543 (5)	0.0664 (6)	0.0495 (5)	−0.0195 (5)	−0.0240 (4)	0.0156 (5)
O1	0.0602 (6)	0.0480 (5)	0.0484 (5)	−0.0020 (4)	0.0003 (4)	−0.0067 (4)
C8	0.0311 (5)	0.0475 (7)	0.0380 (6)	0.0005 (5)	0.0000 (4)	0.0010 (5)
F1	0.0644 (6)	0.0712 (6)	0.0810 (6)	−0.0309 (5)	−0.0030 (5)	0.0207 (5)
C7	0.0310 (5)	0.0482 (6)	0.0372 (6)	−0.0030 (5)	−0.0034 (4)	0.0074 (5)
C11	0.0361 (5)	0.0410 (6)	0.0376 (6)	0.0002 (4)	0.0015 (4)	0.0046 (5)
C9	0.0314 (5)	0.0411 (6)	0.0366 (5)	−0.0003 (4)	−0.0036 (4)	0.0034 (5)
C4	0.0291 (5)	0.0487 (6)	0.0333 (5)	−0.0018 (4)	−0.0031 (4)	0.0065 (5)
C15	0.0362 (6)	0.0545 (7)	0.0530 (7)	−0.0033 (5)	−0.0068 (5)	0.0078 (6)
C10	0.0369 (6)	0.0432 (6)	0.0372 (6)	−0.0014 (5)	−0.0047 (5)	0.0052 (5)
C14	0.0420 (6)	0.0451 (7)	0.0539 (7)	−0.0085 (5)	0.0047 (5)	0.0049 (6)
C5	0.0282 (5)	0.0493 (7)	0.0414 (6)	−0.0010 (5)	−0.0013 (4)	0.0077 (5)
C16	0.0383 (6)	0.0495 (7)	0.0481 (7)	0.0010 (5)	−0.0037 (5)	0.0152 (6)
C3	0.0416 (6)	0.0738 (9)	0.0413 (6)	−0.0025 (6)	0.0023 (5)	0.0203 (6)
C13	0.0590 (8)	0.0550 (8)	0.0468 (7)	−0.0084 (6)	−0.0006 (6)	0.0184 (6)
C12	0.0471 (7)	0.0560 (7)	0.0389 (6)	−0.0066 (6)	−0.0049 (5)	0.0098 (5)
C6	0.0469 (7)	0.0505 (7)	0.0573 (8)	0.0049 (6)	−0.0003 (6)	0.0047 (6)
C2	0.0610 (8)	0.0695 (10)	0.0644 (9)	−0.0146 (7)	−0.0041 (7)	0.0339 (8)
C1	0.0693 (9)	0.0531 (8)	0.0738 (10)	0.0022 (7)	−0.0094 (8)	0.0216 (7)

### Geometric parameters (Å, °)

**Table:**

O4—C7	1.3500 (15)	C14—C13	1.371 (2)
O4—C4	1.4458 (13)	C5—C6	1.5208 (19)
O3—C8	1.3463 (16)	C5—H5A	0.9700
O3—C4	1.4440 (15)	C5—H5B	0.9700
O2—C7	1.1972 (14)	C16—H16A	0.9300
O1—C8	1.2008 (15)	C3—C2	1.524 (2)
C8—C9	1.4830 (16)	C3—H3A	0.9700
F1—C14	1.3511 (15)	C3—H3B	0.9700
C7—C9	1.4872 (16)	C13—C12	1.3845 (19)
C11—C12	1.3922 (17)	C13—H13A	0.9300
C11—C16	1.3948 (17)	C12—H12A	0.9300
C11—C10	1.4635 (17)	C6—C1	1.519 (2)
C9—C10	1.3411 (17)	C6—H6A	0.9700
C4—C3	1.5092 (18)	C6—H6B	0.9700
C4—C5	1.5138 (16)	C2—C1	1.517 (2)
C15—C14	1.365 (2)	C2—H2A	0.9700
C15—C16	1.3827 (18)	C2—H2B	0.9700
C15—H15A	0.9300	C1—H1A	0.9700
C10—H10A	0.9300	C1—H1B	0.9700
			
C7—O4—C4	118.46 (9)	H5A—C5—H5B	108.0
C8—O3—C4	118.15 (9)	C15—C16—C11	120.91 (12)
O1—C8—O3	119.04 (11)	C15—C16—H16A	119.5
O1—C8—C9	124.97 (12)	C11—C16—H16A	119.5
O3—C8—C9	115.69 (10)	C4—C3—C2	112.08 (12)
O2—C7—O4	119.51 (11)	C4—C3—H3A	109.2
O2—C7—C9	125.10 (12)	C2—C3—H3A	109.2
O4—C7—C9	115.36 (9)	C4—C3—H3B	109.2
C12—C11—C16	118.66 (11)	C2—C3—H3B	109.2
C12—C11—C10	118.86 (11)	H3A—C3—H3B	107.9
C16—C11—C10	122.37 (11)	C14—C13—C12	118.29 (13)
C10—C9—C8	125.84 (11)	C14—C13—H13A	120.9
C10—C9—C7	118.84 (10)	C12—C13—H13A	120.9
C8—C9—C7	115.16 (11)	C13—C12—C11	120.75 (12)
O3—C4—O4	108.60 (10)	C13—C12—H12A	119.6
O3—C4—C3	106.43 (10)	C11—C12—H12A	119.6
O4—C4—C3	106.66 (10)	C1—C6—C5	111.29 (12)
O3—C4—C5	110.87 (10)	C1—C6—H6A	109.4
O4—C4—C5	111.68 (9)	C5—C6—H6A	109.4
C3—C4—C5	112.35 (11)	C1—C6—H6B	109.4
C14—C15—C16	118.30 (12)	C5—C6—H6B	109.4
C14—C15—H15A	120.8	H6A—C6—H6B	108.0
C16—C15—H15A	120.8	C1—C2—C3	111.48 (13)
C9—C10—C11	128.90 (11)	C1—C2—H2A	109.3
C9—C10—H10A	115.6	C3—C2—H2A	109.3
C11—C10—H10A	115.6	C1—C2—H2B	109.3
F1—C14—C15	118.41 (12)	C3—C2—H2B	109.3
F1—C14—C13	118.51 (13)	H2A—C2—H2B	108.0
C15—C14—C13	123.08 (12)	C2—C1—C6	110.99 (13)
C4—C5—C6	111.58 (11)	C2—C1—H1A	109.4
C4—C5—H5A	109.3	C6—C1—H1A	109.4
C6—C5—H5A	109.3	C2—C1—H1B	109.4
C4—C5—H5B	109.3	C6—C1—H1B	109.4
C6—C5—H5B	109.3	H1A—C1—H1B	108.0

### Hydrogen-bond geometry (Å, °)

**Table:**

*D*—H···*A*	*D*—H	H···*A*	*D*···*A*	*D*—H···*A*
C12—H12A···O2^i^	0.93	2.47	3.3405 (17)	156
C10—H10A···O2	0.93	2.54	2.874 (2)	101

Symmetry codes: (i) −*x*+1, −*y*, −*z*+2.
